# The efficacy of telephone follow-up frequencies on clinical parameters post non-surgical periodontal therapy: a randomized controlled trial

**DOI:** 10.3389/froh.2025.1568252

**Published:** 2025-04-10

**Authors:** Yinghui He, Feng Tang, Ruoyi Liao, Chun Hu, Hongyu Liu

**Affiliations:** ^1^ Department of Pharmacy, The First Hospital of Hunan University of Chinese Medicine, Changsha, China; ^2^ Department of Nursing, The First Hospital of Hunan University of Chinese Medicine, Changsha, China; ^3^ Department of Stomatology, The First Hospital of Hunan University of Chinese Medicine, Changsha, China

**Keywords:** periodontitis, telephone follow-up, non-surgical periodontal therapy, periodontal parameters, randomized controlled trial

## Abstract

**Objectives:**

This randomized controlled trial aimed to investigate the impact of different telephone follow-up frequencies on periodontal clinical parameters after non-surgical periodontal therapy.

**Materials and methods:**

Patients with Stage II–IV periodontitis were enrolled and randomly assigned to high-frequency (once every 2 weeks), medium-frequency (once a month), and low-frequency (once in 3 months) follow-up groups. All patients received standard non-surgical periodontal treatment. The full mouth probing depth (PD), clinical attachment loss (CAL), gingival index (GI), and plaque index (PI) were evaluated at baseline, after treatment (T1) and post treatment 3 months (T2).

**Results:**

From T1 to T2, the high-frequency follow-up group had significant reduced in PD (*p* = 0.03), improved in GI (*p* = 0.04) and PI (*p* = 0.03) compared with the medium and low-frequency groups. There was no significant difference in PD, GI, and PI between the medium-frequency group and the low-frequency group. No statistical difference was found in CAL among the three groups.

**Conclusion:**

More frequent telephone follow-up helps maintain and enhance non-surgical periodontal therapy effects.

## Introduction

Periodontitis is a prevalent chronic inflammatory disease of the oral cavity that not only compromises oral health but also has far-reaching implications for systemic well-being (1–4). It is characterized by the destruction of the periodontal supporting tissues, including the gingiva, periodontal ligament, cementum, and alveolar bone (5). The global burden of periodontitis is substantial, affecting a large proportion of the population across different age groups and socioeconomic strata (6, 7).

Non-surgical periodontal therapy represents the cornerstone of periodontitis treatment. It encompasses a series of procedures designed to eliminate the etiological factors, primarily dental plaque biofilm and its by-products, and to arrest the progression of the disease. These procedures include meticulous oral hygiene instruction and thorough scaling and root planing (8, 9). Despite the effectiveness of non-surgical periodontal therapy in reducing inflammation and improving periodontal health in the short-term, the long-term success of treatment is contingent upon patients' ability to maintain optimal oral hygiene practices and adhere to regular follow-up appointments (10).

Telephone follow-up has emerged as a valuable tool in the management of chronic diseases, offering a convenient, cost-effective, and accessible means of patient communication. By providing timely reminders, reinforcement of health education, and addressing patients' concerns, telephone follow-up can enhance patients' understanding of their condition, improve treatment adherence, and ultimately lead to better health outcomes (11). In the context of periodontal treatment, telephone follow-up can play a crucial role in promoting patients' self-care behaviors, such as regular brushing, flossing, and proper use of oral hygiene aids, as well as ensuring their compliance with recommended follow-up schedules (12). However, the optimal frequency of telephone follow-up after periodontal therapy remains a subject of debate. Insufficient follow-up may result in patients reverting to poor oral hygiene habits and neglecting their periodontal health, while overly frequent follow-up may cause patient fatigue and non-compliance. Therefore, determining the most appropriate telephone follow-up frequency is essential for maximizing the benefits of periodontal treatment and improving long-term patient outcome.

## Materials and methods

### Study design

This was a parallel-controlled, single-blind (blinded for examiner and data analyst) randomized controlled clinical trial. The study was designed to compare the effects of different telephone follow-up frequencies on periodontal clinical parameters 3 months after non-surgical periodontal therapy. The trial was conducted in accordance with the principles of the Declaration of Helsinki and Good Clinical Practice guidelines.

### Patient selection

The study population consisted of patients with Stage II–IV periodontitis who presented to the Department of Stomatology of The First Hospital of Hunan University of Chinese Medicine between April 2024 and September 2024. To improve the calibration efficiency of periodontal clinical parameter measurement, patients with Stage I periodontitis were not included in this study, as they are likely to cause measurement errors. Patients were diagnosed based on the consensus report on the classification of periodontal and peri-implant diseases and conditions in 2018 (13). Aged between 18 and 60 years old. Minimum number of teeth ≥20. All patients had to have more than two non-adjacent sites with interdental probing attachment loss ≥3 mm, more than two non-adjacent sites with probing pocket depth ≥5 mm, and radiological bone loss ≥15%.

The exclusion criteria encompass the following: long-term alcohol abuse; women who are pregnant or lactating; those who have received antibiotic therapy or periodontal therapy within the last 6 months; patients with systemic diseases (such as hypertension, diabetes, hyperlipidemia, respiratory disorders, malignant tumors, liver or renal insufficiency, etc.); individuals undergoing orthodontic treatment; those who have undergone head and neck radiotherapy or chemotherapy; and those who are unable to sign the informed consent.

### Randomization and grouping

A computer-generated random number table was used to randomly allocate the eligible patients into three groups: the high-frequency follow-up group (High), the medium-frequency follow-up group (Medium), and the low-frequency follow-up group (Low), at a ratio of 1:1:1. The random number table was generated by a statistician who was not involved in the patient recruitment, treatment, or follow-up processes. The generated random allocation sequence was placed into sequentially coded, sealed, opaque envelopes. The grouping information was concealed from the patients and the treating clinicians until the baseline assessments were completed. Upon determining the eligibility of participants, researchers opened the envelopes in sequence and assigned the participants to the corresponding trial groups. This randomization process ensured that each patient had an equal chance of being assigned to any of the three groups, minimizing selection bias.

### Non-surgical periodontal therapy

Due to the work and study schedules of the patients, we were unable to ensure that each patient received exactly the same number of outpatient appointments and treatments. However, we ensured that all patients received comprehensive non-surgical periodontal therapy, including oral hygiene instruction, supragingival scaling (PIEZON® 150, EMS, Switzerland), and subgingival root planing (Gracey curettes, Hu-Friedy, USA). To avoid introducing confounding factors, the treatment process exclusively utilized 0.12% chlorhexidine and 3% hydrogen peroxide for irrigation. No antibiotics or laser therapy were administered during the intervention. Treatments were delivered by a team of experienced periodontal specialists who had undergone standardized training to ensure consistency in treatment procedures. To rule out the impact of this stage on the baseline periodontal clinical parameters of the three groups, we measured the periodontal clinical parameters and conduct inter-group comparisons one month following the conclusion of non-surgical periodontal therapy, before the start of the telephone follow-up (T1).

### Telephone follow-up

Patients in the high-frequency follow-up group received telephone calls every 2 weeks after the completion of non-surgical periodontal therapy. Each telephone call was conducted by a trained nurse who was familiar with periodontal disease management. The duration of each call was approximately 10–15 min. During the calls, the nurse reinforced the oral hygiene instructions provided during the initial visit, asked about the patients' oral hygiene practices, such as the frequency of brushing, flossing, and the use of interdental brushes, and provided personalized advice based on the patients' responses. The nurse also reminded the patients of their upcoming follow-up appointments, addressed any concerns or questions the patients had regarding their periodontal health or treatment, and provided encouragement and motivation to maintain good oral hygiene habits.

Patients in the medium-frequency follow-up group received telephone calls once a month after treatment. The content and duration of the telephone calls were similar to those in the high-frequency follow-up group. The medium-frequency follow-up was designed to provide a balance between regular communication and minimizing patient burden, aiming to reinforce oral hygiene instructions and maintain patient engagement without causing excessive disruption.

Patients in the low-frequency follow-up group received a single telephone call 6 weeks after treatment (once in 3 months after treatment). The nurse followed the same protocol as in the other two groups during the call, but with a less frequent schedule. This group was included to assess the impact of less intensive follow-up on patient outcomes and to determine whether a lower frequency of communication could still have a beneficial effect on periodontal health.

### Periodontal parameters measurement

Each patient was examined and evaluated by the same calibrated periodontist. Full mouth probing depth (PD), clinical attachment loss (CAL), gingival index (GI) and plaque index (PI) were recorded using periodontal probe (PCPUNC15, Hu-Friedy, USA) at three time points, namely before the non-surgical periodontal therapy (T0), before the commencement of the telephone follow-up phase (T1), and 3 months post-T1 (T2). The PI was measured using the Fischman Plaque Index system, which assesses the amount of plaque on the tooth surface (15). The GI was measured using the Löe and Silness Gingival Index system, which evaluates the degree of gingival inflammation (14).

### Examiner calibration

Five patients were chosen from among the study participants for calibration. PD, CAL, GI and PI were measured twice, with 2 days between the examinations. For PD, the percentage of agreement within ±1 mm between repeated measurements was 98%. For CAL, the percentage of agreement within ±1 mm between repeated measurements was 97.5%. For GI, the percentage of agreement within ±1 between repeated measurements was 98.5%. For PI, the percentage of agreement within ±1 between repeated measurements was 97%.

### Sample size calculation

Sample size calculation for this study was performed using G*Power 3.1.9.7 software (Heinrich-Heine-Universität Düsseldorf, Germany), which was based on the data gathered in a pilot study. The primary outcome of our study was the change in full mouth mean PD from T1 to T2.The sample size analysis was determined by taking into account three groups of participants, an expected standard deviation of 0.5, a two-sided significance level of 0.05, and a power level of 80%. It was established that a minimum sample size of 40 patients per group was required for adequate statistical power.

### Statistical analysis

Statistical analysis was performed using GraphPad Prism 10 (La Jolla, CA). For baseline characteristics, one-way ANOVA (with Levene's test for variance homogeneity check, using Welch's ANOVA if needed) was used for continuous variables like age, PD, CAL, GI and PI, and the chi-square test for categorical variables like gender and smoking status. From T0 to T1 and T1 to T2, paired *t*-tests examined changes within groups for PD, CAL, GI and PI. One-way ANOVA followed by LSD *post-hoc* tests compared these parameters among groups. The significance level was set at *α* = 0.05, with a two-sided test employed to ensure statistical rigor.

## Results

### Demographics and baseline clinical parameters

**Figure 1:**
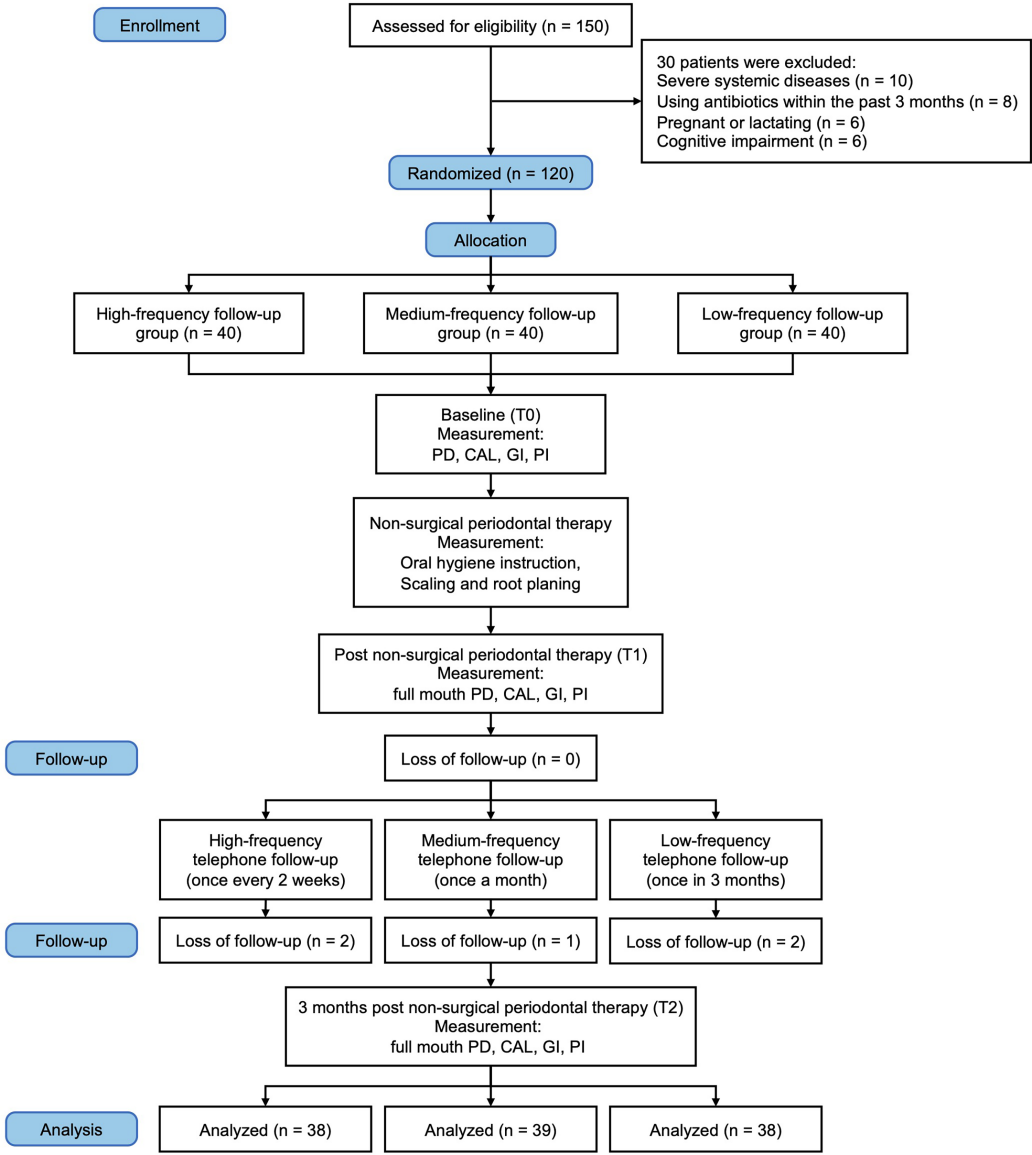
CONSORT flow diagram.

A total of 150 patients were initially screened for eligibility. Of these, 30 patients were excluded due to various reasons: 10 patients had severe systemic diseases, 8 patients had used antibiotics within the past 3 months, 6 patients were pregnant or lactating, and 6 patients had cognitive impairment. Thus, 120 patients were enrolled in the study and randomly assigned to the high-frequency (*n* = 40), medium-frequency (*n* = 40), and low-frequency (*n* = 40) follow-up groups. During the course of the study, 5 patients dropped out: 2 from the high-frequency group (due to relocation and personal reasons), 1 from the medium-frequency group (due to non-compliance with the treatment protocol), and 2 from the low-frequency group (due to loss of interest). The final analysis was conducted on 115 patients (38 in the high-frequency group, 39 in the medium-frequency group, and 38 in the low-frequency group) (Figure 1).

The baseline characteristics of the patients in the three groups are presented in Table 1. There were no significant differences in age, gender distribution, smoking status, or baseline periodontal parameters (PD, CAL, GI and PI) among the three groups (*p* > 0.05). This indicates that the randomization process was successful in creating comparable groups at the start of the study.

**Table 1:** Baseline characteristics of patients at T0.

Variables	Telephone follow-up frequency			*p* value^a^
	High (*n* = 38)	Medium (*n* = 39)	Low (*n* = 38)	
Age (years, mean ± SD)	37.8 ± 7.5	36.9 ± 8.1	38.2 ± 7.9	0.721
Gender (Male/Female)	20/18	21/18	19/19	0.912
Smoking Status (Smoker/Non-smoker)	12/26	11/28	13/25	0.885
PD (mm, mean ± SD)	5.20 ± 0.80	5.18 ± 0.85	5.22 ± 0.82	0.967
CAL (mm, mean ± SD)	2.83 ± 0.60	2.85 ± 0.65	2.82 ± 0.62	0.923
GI (mean ± SD)	2.03 ± 0.28	2.05 ± 0.30	2.04 ± 0.27	0.945
PI (mean ± SD)	2.23 ± 0.32	2.20 ± 0.35	2.25 ± 0.30	0.843

PD, probing depth; CAL, clinical attachment loss; GI, gingival index; PI, plaque index; SD, standard deviation.

^a^ One-way ANOVA was used for continuous variables like age, PD, CAL, GI and PI, and the chi-square test for categorical variables like gender and smoking status.

### Changes in periodontal parameters from T0 to T1

At T1, one month following the conclusion of non-surgical periodontal therapy, before the commencement of the telephone follow-up phase, the mean PD at T1 was 4.20 ± 0.70 mm in the high-frequency follow-up group, 4.30 ± 0.75 mm in the medium-frequency follow-up group, and 4.35 ± 0.80 mm in the low-frequency follow-up group. Paired *t*-tests demonstrated significant reductions in PD from T0 to T1 within each group (*p* < 0.05 for all). ANOVA did not reveal any significant differences among the groups at T1 (*F* = 1.12, *p* = 0.33).

The mean CAL at T1 was 2.30 ± 0.50 mm in the high-frequency follow-up group, 2.35 ± 0.55 mm in the medium-frequency follow-up group, and 2.40 ± 0.60 mm in the low-frequency follow-up group. Paired *t*-tests showed significant reductions in CAL from T0 to T1 within each group (*p* < 0.05 for all). ANOVA found no significant differences among the groups at T1 (*F* = 1.45, *p* = 0.24).

The mean GI at T1 was 1.30 ± 0.25 in the high-frequency follow-up group, 1.35 ± 0.28 in the medium-frequency follow-up group, and 1.40 ± 0.30 in the low-frequency follow-up group. Paired *t*-tests indicated significant improvements in GI from T0 to T1 within each group (*p* < 0.05 for all). ANOVA showed no significant difference among the groups at T1 (*F* = 1.56, *p* = 0.21).

The mean PI in the high-frequency follow-up group was 1.60 ± 0.30, in the medium-frequency follow-up group was 1.65 ± 0.32, and in the low frequency follow-up group was 1.70 ± 0.33. Paired *t*-tests were used to compare the changes in PI from T0 to T1 within each group. The results showed that there was a significant improvement in PI from T0 to T1 in all groups (*p* < 0.05 for all). However, one-way ANOVA showed no significant difference among the three groups at T1 (*F* = 1.23, *p* = 0.30). Detailed data can be found in Table 2.

**Table 2:** Periodontal parameters of patients at T1.

Variables	Telephone follow-up frequency						*F* value^b^	*p* value^b^
	High (*n* = 38)		Medium (*n* = 39)		Low (*n* = 38)			
	Mean ± SD	*p* value^a^	Mean ± SD	*p* value^a^	Mean ± SD	*p* value^a^		
PD (mm)	4.20 ± 0.70	<0.05	4.30 ± 0.75	<0.05	4.35 ± 0.80	<0.05	1.12	0.33
CAL (mm)	2.30 ± 0.50	<0.05	2.35 ± 0.55	<0.05	2.40 ± 0.60	<0.05	1.45	0.24
GI	1.30 ± 0.25	<0.05	1.35 ± 0.28	<0.05	1.40 ± 0.30	<0.05	1.56	0.21
PI	1.60 ± 0.30	<0.05	1.65 ± 0.32	<0.05	1.70 ± 0.33	<0.05	1.23	0.30

PD, probing depth; CAL, clinical attachment loss; GI, gingival index; PI, plaque index; SD, standard deviation.

^a^ Paired *t*-tests from T0 to T1.

^b^ One-way ANOVA followed by LSD *post-hoc* tests.

### Changes in periodontal clinical parameters from T1 to T2

**Figure 2:**
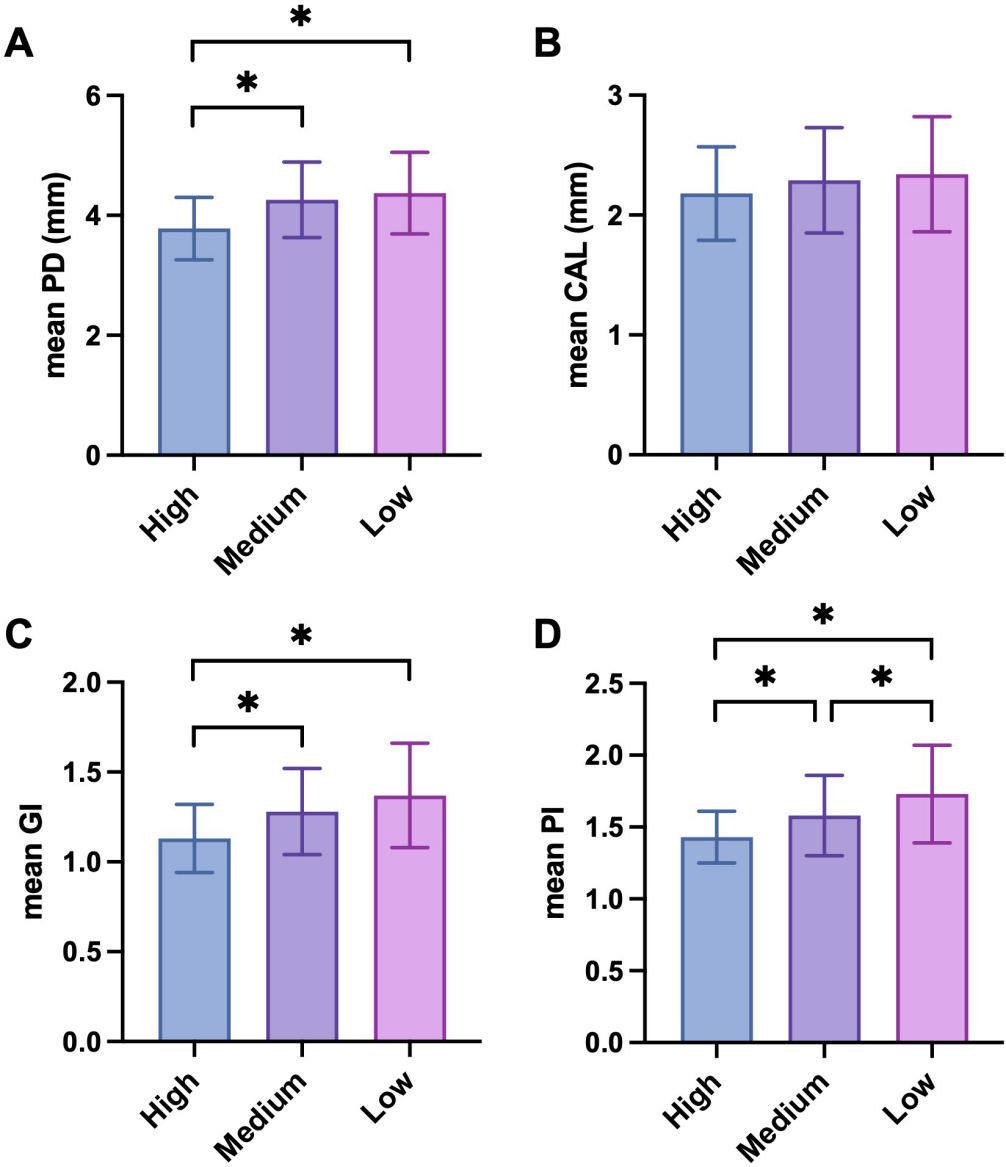
Periodontal parameters among different telephone follow-up frequency groups at 3 months after non-surgical periodontal treatment (T2). **(A)** Mean PD; **(B)** mean CAL; **(C)** mean GI; **(D)** mean PI. PD, probing depth; CAL, clinical attachment loss; GI, gingival index; PI, plaque index. *n* = 38 (High), 39 (Medium) and 38 (Low). Differences were assessed via one-way ANOVA followed by LSD *post-hoc* tests. *p < 0.05; **p < 0.01 and ***p < 0.001.

At T2 (3 months after T1), the mean PD at T2 was 3.78 ± 0.52 mm in the high-frequency follow-up group, 4.23 ± 0.63 mm in the medium-frequency follow-up group, and 4.37 ± 0.68 mm in the low-frequency follow-up group. Paired *t*-tests demonstrated that the high-frequency follow-up group had a statistically significant reduction in PD from T1 to T2 (*p* = 0.03), the medium-frequency follow-up group had no statistically significant change from T1 to T2 (*p* = 0.30), and the low-frequency follow-up group had a small but not statistically significant increase in PD from T1 to T2 (*p* = 0.08). ANOVA among the three groups at T2 was significant (*F* = 9.56, *p* < 0.001), and pairwise comparisons using the LSD-t test showed that the high-frequency follow-up group had a significantly lower PD compared to the medium-frequency and low-frequency follow-up groups (*p* < 0.05), while the medium-frequency follow-up group and the low-frequency follow-up group had no significant difference in PD (*p* > 0.05) (Figure 2A).

The mean CAL at T2 was 2.18 ± 0.39 mm in the high-frequency follow-up group, 2.29 ± 0.44 mm in the medium-frequency follow-up group, and 2.23 ± 0.48 mm in the low-frequency follow-up group. Paired *t*-tests showed that the high-frequency follow-up group had no statistically reduction in CAL from T1 to T2 (*p* = 0.07), the medium-frequency follow-up group had no statistically significant difference from T1 to T2 (*p* = 0.22), and the low-frequency follow-up group had a small but not statistically significant increase in CAL from T1 to T2 (*p* = 0.09). ANOVA at T2 was not significant (*F* = 2.15, *p* = 0.12), indicating no statistically significant differences in CAL among the three groups (Figure 2B).

The mean GI at T2 was 1.13 ± 0.19 in the high-frequency follow-up group, 1.28 ± 0.24 in the medium-frequency follow-up group, and 1.37 ± 0.29 in the low-frequency follow-up group. Paired *t*-tests indicated that the high-frequency follow-up group had a statistically significant improvement in GI from T1 to T2 (*p* = 0.04), the medium-frequency follow-up group had no statistically significant difference from T1 to T2 (*p* = 0.20), and the low-frequency follow-up group also had no statistically significant change in GI from T1 to T2 (*p* = 0.09). ANOVA at T2 was significant (*F* = 8.32, *p* < 0.001), and *post-hoc* analysis using the LSD-t test showed that the high-frequency follow-up group had a significantly lower GI compared to the other two groups (*p* < 0.05), while the medium-frequency follow-up group and the low-frequency follow-up group had no significant difference in GI (*p* > 0.05) (Figure 2C).

The mean PI in the high-frequency follow-up group was 1.43 ± 0.18, in the medium-frequency follow-up group was 1.58 ± 0.28, and in the low-frequency follow-up group was 1.73 ± 0.34. Paired *t*-tests showed that the high-frequency follow-up group had a statistically significant improvement in PLI from T1 to T2 (*p* = 0.03), the medium-frequency follow-up group had no statistically significant difference from T1 to T2 (*p* = 0.28), and the low-frequency follow-up group had a statistically significant worsening in PI from T1 to T2 (*p* = 0.04). One-way ANOVA among the three groups at T2 was significant (*F* = 10.26, *p* < 0.001), and *post-hoc* pairwise comparisons using the LSD-t test revealed that the high-frequency follow-up group had a significantly lower PI compared to the medium-frequency and low-frequency follow-up groups (*p* < 0.05 for both comparisons), and the medium-frequency follow-up group had a significantly lower PI compared to the low-frequency follow-up group (*p* < 0.05) (Figure 2D). Detailed data can be found in Table 3.

**Table 3:** Periodontal parameters of patients at T2.

Variables	Telephone follow-up frequency						*F* value^b^	*p* value^b^
	High (*n* = 38)		Medium (*n* = 39)		Low (*n* = 38)			
	Mean ± SD	*p* value^a^	Mean ± SD	*p* value^a^	Mean ± SD	*p* value^a^		
PD (mm)	3.78 ± 0.52	<0.05	4.23 ± 0.63	0.30	4.37 ± 0.68	0.08	9.56	<0.001
CAL (mm)	2.18 ± 0.39	0.07	2.29 ± 0.44	0.22	2.23 ± 0.48	0.09	2.15	0.12
GI	1.13 ± 0.19	<0.05	1.28 ± 0.24	0.20	1.37 ± 0.29	0.09	8.32	<0.001
PI	1.43 ± 0.18	<0.05	1.58 ± 0.28	0.28	1.73 ± 0.34	<0.05	10.26	<0.001

PD, probing depth; CAL, clinical attachment loss; GI, gingival index; PI, plaque index; SD, standard deviation.

^a^ Paired *t*-tests from T1 to T2.

^b^ One-way ANOVA followed by LSD *post-hoc* tests.

## Discussion

The results from T0 to T1 suggest that all groups showed significant improvements in periodontal clinical parameters after the non-surgical periodontal therapy, which is expected as the treatment aims to address the initial periodontal problems. The lack of significant differences among the groups at T1 indicates that the initial treatment effect was similar regardless of the follow-up frequency grouping. Meanwhile, this also indicates that the different treatment and appointment frequencies of patients during non-surgical periodontal therapy have no impact on the baseline before telephone follow-up (T1). This phenomenon can be attributed to the fact that the immediate post-treatment period may primarily be influenced by the direct impact of the non-surgical periodontal therapy, which acts uniformly across different follow-up groups in the short term. The removal of plaque and calculus during the treatment likely leads to a reduction in inflammation and improvement in various clinical parameters, as observed in similar studies (16, 17). These studies have demonstrated that the initial treatment phase usually results in a generalized improvement in periodontal health, regardless of subsequent follow-up strategies, due to the mechanical and chemical effects of the basic treatment modalities.

From T1 to T2, the different patterns of changes in the three groups highlight the impact of follow-up frequency over time. The high-frequency follow-up group's small but significant improvements in PD, GI and PI suggest that more frequent follow-ups can help maintain and slightly enhance the treatment effect. This could be because frequent follow-ups allow for timely identification and intervention of potential problems, such as the recurrence of plaque accumulation or gingival inflammation. The worsening PI in the low-frequency follow-up group implies that infrequent follow-ups may lead to a decline in periodontal health, possibly due to patients' decreased adherence to oral hygiene and treatment protocols without adequate reinforcement. Similar findings have been documented by Manresa et al. (18), who emphasized that insufficient follow-up frequency could lead to a relapse of periodontal disease, suggesting that patients in low-frequency follow-up groups might have more difficulty in maintaining a proper oral care routine, resulting in the reaccumulation of plaque and worsening of clinical parameters.

In the context of this study, the use of telephone follow-up has several unique characteristics. Firstly, telephone follow-up offers a convenient and cost-effective means of maintaining communication with patients. As noted by Dixon et al. (19), it eliminates the need for patients to travel to the clinic for every follow-up appointment, reducing time and financial burdens. This convenience may increase patient participation in follow-up programs, especially for those with limited mobility or busy schedules.

However, telephone follow-up also has limitations. Visual assessment of periodontal conditions is not possible over the phone. As a result, periodontists rely solely on patients' self-reporting of symptoms and oral hygiene practices. This can lead to inaccuracies, as patients may not accurately describe their oral conditions or may over- or under-report their adherence to oral hygiene instructions. For example, research by Ream et al. (20) found that patients may over-estimate their compliance during telephone interviews. Despite these limitations, telephone follow-up can still be an effective way to reinforce oral hygiene education. Through verbal communication, periodontists can remind patients of proper brushing techniques, the importance of flossing, and the need for regular dental check-ups. In a study by Koh et al. (21), telephone-based oral hygiene education was shown to have a positive impact on patients' knowledge and self-reported oral hygiene behaviors.

The study's limitation is that the follow-up period is limited to 3 months. While significant differences were observed, longer-term follow-up could provide more information on the durability of the effects of different follow-up frequencies. Longer follow-up periods have been recommended by several researchers, such as Guarnieri et al. (22), who found that some periodontal improvements may not be stable beyond 10 years, indicating the importance of extended follow-up. As the study was conducted in a single center, the generalizability of the results may be limited. Multicenter studies with diverse patient populations would enhance the robustness of the findings. Although efforts were made to blind data analysts, complete blinding was not possible, which may have introduced some bias.

## Conclusion

This randomized controlled trial demonstrated distinct patterns of periodontal clinical parameter changes under different telephone follow-up frequencies. At T1, all groups improved without significant differences, indicating the initial effectiveness of treatment. From T1 to T2, the high-frequency follow-up group showed continued improvement in PD, GI and PI, the medium-frequency group remained stable, and the low-frequency group deteriorated in PI. These results indicate that more frequent telephone follow-up can enhance treatment effects. Our findings provide valuable insights into optimizing telephone follow-up strategies for periodontal therapy.

## Data Availability

The original contributions presented in the study are included in the article/Supplementary Material, further inquiries can be directed to the corresponding authors.
